# Recurrent primary intracranial myxofibrosarcoma: a case report and review of the literature

**DOI:** 10.3389/fonc.2026.1755019

**Published:** 2026-01-23

**Authors:** Shuting Wen, Mengqi Tu, Rufang Liao, Huan Li

**Affiliations:** 1 Department of Radiology, Second People’s Hospital of Yuhang District, Hangzhou, China; 2 Department of Radiology, Zhongnan Hospital of Wuhan University, Wuhan, China

**Keywords:** intracranial myxofibrosarcoma, histopathology, non-contiguous recurrence, cerebrospinal fluid dissemination, radiotherapy

## Abstract

Primary intracranial myxofibrosarcoma (MFS) is an exceedingly rare mesenchymal malignancy, with only a few cases reported. We report a 34-year-old male with a right frontal lobe MFS who subsequently developed two non-contiguous recurrences in the right temporal and left frontal lobes. Histopathological examination revealed a stepwise progression from low- to intermediate- to high-grade disease, accompanied by progressively increasing Ki-67 labeling indices. Notably, the pattern of spatially separate recurrences suggests the possibility of cerebrospinal fluid–mediated dissemination. This case highlights the aggressive and heterogeneous nature of intracranial MFS and underscores the importance of long-term, comprehensive follow-up to detect recurrences, particularly at non-contiguous sites.

## Background

Myxofibrosarcoma (MFS), previously considered as the myxoid variant of malignant fibrous histiocytoma (MFH), has been redefined by the World Health Organization (WHO) as a distinct entity distinguished by myxoid matrix, pleomorphic spindle cells, and characteristic curvilinear vasculature. MFS is a rare mesenchymal malignancy, accounting for approximately 5–10% of all soft tissue sarcomas (STS) (1). It most commonly occurs in the extremities of elderly patients. Clinically, MFS is most commonly characterized by a painless, superficial mass. Its infiltrative growth pattern accounts for the high rate of local recurrence (2).

Primary intracranial MFS is exceedingly rare, with only a few cases documented in the literature. Recent genomic studies have demonstrated that copy number alterations and recurrent gene mutations play important roles in the pathogenesis of MFS. Frequently altered genes include TP53, RB1, CDKN2A/B, NF1, NTRK1, MDM2, and PTEN, while additional recurrent mutations have been identified in GNAS, ATRX, KRAS, CCND1, and JAK1 (3). These findings highlight the molecular heterogeneity of MFS and suggest that genetic alterations in key signaling pathways may contribute to its tumor progression and recurrence.

Here, we report a case of primary intracranial MFS in a 34-year-old male, presenting with two recurrences and progressive histological upgrading. This report underscores the rarity and aggressive clinical behavior of intracranial MFS, highlighting the diagnostic and therapeutic challenges it poses in clinical practice.

## Case report

**Figure 1:**
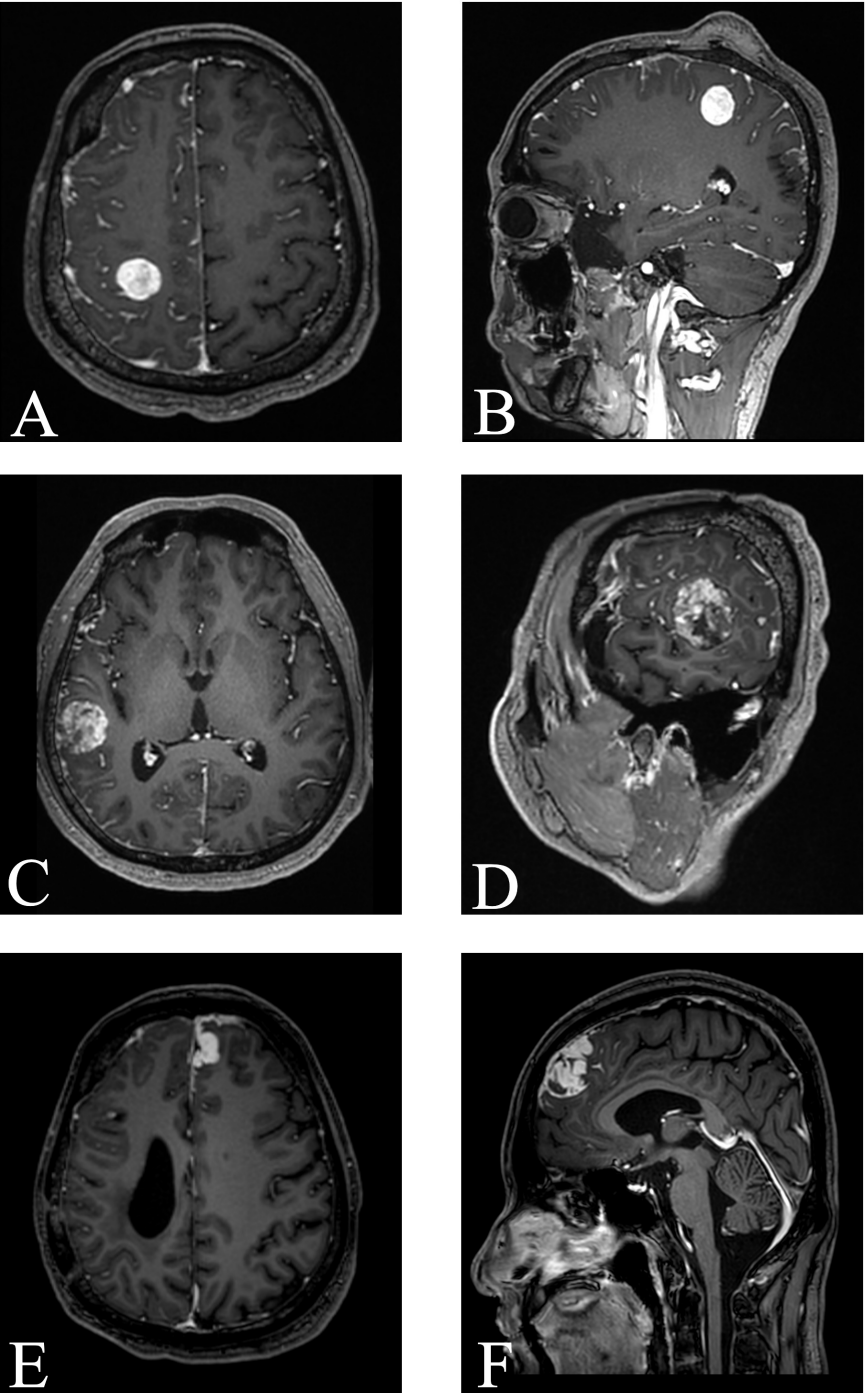
MR enhancement scans obtained before each surgery: **(A, B)** right frontal lesion before the first resection. **(C, D)** right temporal lesion before the second resection. **(E, F)** left frontal lesion before the third resection.

**Figure 2:**
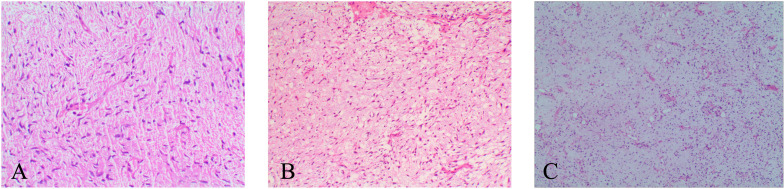
Histopathological features of MFS. [**(A)**, ×100] Tumor cells show moderate cellularity and are embedded in a prominent myxoid stroma. [**(B)**, ×100] Tumor cells show increased cellularity and more pronounced nuclear atypia, with the myxoid stroma still evident. [**(C)**, ×100] Tumor cells show markedly increased cellularity, greater nuclear pleomorphism, reduced myxoid stroma, and focal necrosis.

A 34-year-old male presented with transient left-sided limb numbness and mild weakness. He reported no other associated symptoms, such as pain, nausea, or vomiting. Neurological examination revealed no significant abnormalities. The patient had no relevant surgical history and no known family history of tumors. He denied any history of chronic diseases or infectious diseases. Contrast-enhanced magnetic resonance imaging (MRI) demonstrated an ovoid intra-axial mass in the right frontal lobe (precentral gyrus) with significant enhancement and associated extensive leptomeningeal enhancement in the right frontoparietal-temporal region (**Figures 1A, B**). The patient underwent a right frontal craniotomy. Intraoperatively, the lesion appeared dark in color and was highly vascularized, with a marked leptomeningeal proliferation on the cortical surface. The tumor was resected in multiple fragments and completely removed. Under light microscopy, the tumor cells were spindle-shaped, of moderate cellularity, within a myxoid stroma, accompanied by numerous thin-walled, curvilinear vessels (**Figure 2A**). The results of immunohistochemistry were as follows: MDM2 (+), SATB2 (+), GFAP (-), Olig-2 (-), CD34 (-), Desmin (-), SMA (-), S-100 (-), SSTR2 (-), EMA (-) and MyoD1 (-), and Ki-67 (+ about 20%) (**Table 1**). Based on these findings, the lesion was diagnosed as low-grade MFS. This grading was determined through an integrated histopathological assessment of tumor cellularity, nuclear atypia, mitotic activity, and microvascular proliferation. The Ki-67 proliferation index was considered an adjunct marker of proliferative activity. MDM2 immunostaining was performed primarily to exclude dedifferentiated liposarcoma. Despite its positivity, the finding was considered nonspecific in the absence of characteristic histological features. Likewise, although SATB2 expression was observed, no evidence of osteoid production or malignant osteogenic tumor cells was identified, which excluded the diagnosis of osteosarcoma. Next-generation sequencing (NGS) analysis identified mutations in NRAS, BAP1, KDR, PIK3CA, and BLMT.

**Table 1:** Immunohistochemical makers of low-, intermediate-, and high-grade MFS.

Diagnosis/marker	Low-grade MFS	Intermediate-grade MFS	High-grade MFS
CD34	–	N/A	+
Olig-2	–	N/A	–
S-100	–	N/A	–
Desmin	–	–	–
SMA	–	–	–
MDM2	+	N/A	N/A
SATB2	+	N/A	N/A
GFAP	–	N/A	–
SSTR2	–	–	N/A
MyoD1	N/A	N/A	N/A
IDH1R132H	–	N/A	N/A
EMA	–	–	–
BAP1	N/A	+	N/A
SS18-SSX	N/A	–	N/A
CK	N/A	N/A	–
VIMENTIN	N/A	N/A	+
P16	+	N/A	+
PR	–	N/A	–
STAT6	–	N/A	–
INI-1	+	N/A	+
H3K27Me3	N/A	N/A	+
Ki-67(%)	20	30	40

MFS, Myxofibrosarcoma; +, positive; -, negative; N/A, not available.

Considering the poor sensitivity of this tumor to chemotherapy, postoperative adjuvant radiotherapy was chosen as the primary adjuvant treatment. Radiotherapy was initially performed at another hospital, where detailed radiotherapy records were unavailable. The patient developed a wound infection after 15 sessions, which necessitated the interruption of radiotherapy and appropriate wound care. Ultimately, the patient completed a total of 32 radiotherapy sessions. Six months later, the patient underwent cranioplasty at our institution and recovered well postoperatively. Follow-up evaluations, including clinical assessment and imaging, demonstrated a stable condition without evidence of tumor recurrence.

Thirteen months after the initial resection, follow-up MRI revealed a new right temporal intra-axial lesion with heterogeneous, marked contrast enhancement and adjacent leptomeningeal involvement (**Figures 1C, D**). A second surgical resection was performed under 5-ALA fluorescence guidance. Intraoperatively, the tumor was located subcortically, appeared pale red, and was well-demarcated. Resection proceeded from the tumor center outward, achieving gross total removal. Histopathological examination confirmed an intermediate-grade MFS (**Figure 2B**). Immunohistochemistry demonstrated retained nuclear BAP1 expression, EMA (−), Desmin (−), SMA (−), SSTR2 (−), SS18-SSX (−), and a Ki-67 index of approximately 30% (**Table 1**). The retained nuclear BAP1 supports exclusion of BAP1-associated tumors such as mesothelioma and provides supplementary information for the differential diagnosis. Postoperatively, the patient underwent localized adjuvant radiotherapy directed at the right temporal resection cavity and adjacent leptomeningeal enhancement, with a total prescribed dose of 56 Gy to the PTV and 50 Gy to the GTV/CTV delivered in 28 fractions.

Nineteen months after the second resection, follow-up MRI demonstrated a predominant left frontal intra-axial lesion with contiguous leptomeningeal and dural enhancement, along with a smaller right frontal lesion (**Figures 1E, F**). Surgical resection was performed, with the right frontal lesion removed first, followed by the left frontal lesion. Intraoperatively, the tumor demonstrated moderate vascularity, was well-circumscribed, and was resected en bloc. Endoscopic inspection revealed no additional abnormalities. Histopathological examination confirmed a high-grade MFS (**Figure 2C**). Immunohistochemistry showed positivity for CD34, P16, and Vimentin, negativity for CK, SMA, Desmin, EMA, S-100, GFAP, Olig-2, SSTR2, PR, and STAT6, and retained expression of INI-1 and H3K27Me3 (**Table 1**). The retained nuclear H3K27Me3 supports the exclusion of malignant peripheral nerve sheath tumor (MPNST). The Ki-67 proliferation index was approximately 40%. Postoperatively, the patient underwent localized adjuvant radiotherapy targeting the frontal resection cavity and adjacent leptomeningeal enhancement, with a total dose of 60 Gy delivered in 24 fractions.

**Figure 3:**
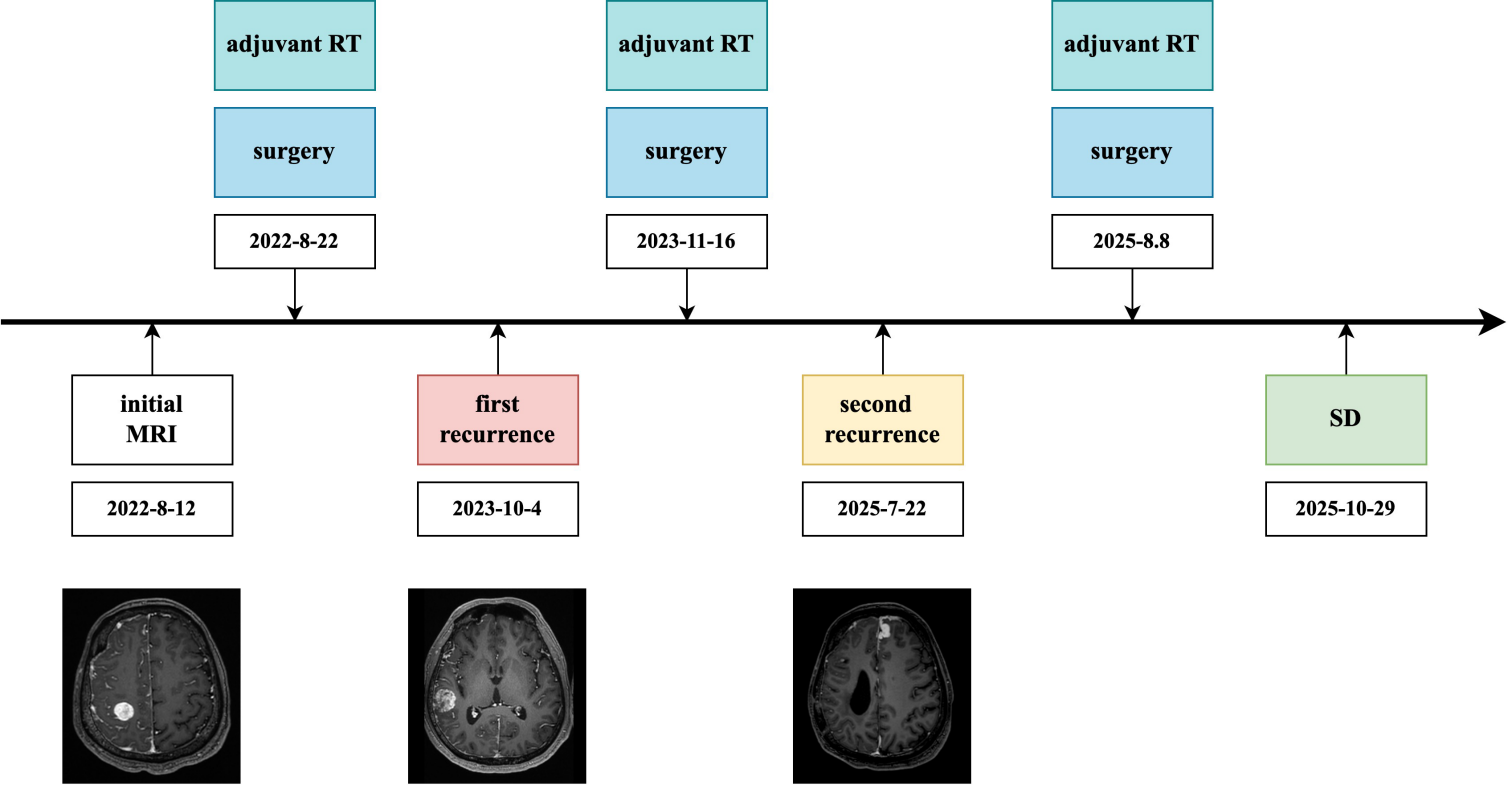
Clinical timeline of the patient, illustrating the sequence of initial presentation, surgical resections, adjuvant radiotherapy, recurrences, and follow-up outcomes over the disease course. SD, stable disease.

At the two-month postoperative follow-up, imaging demonstrated stable disease without evidence of tumor recurrence. The patient reported no significant neurological deficits and was able to perform daily activities independently. A complete timeline of this case can be seen in **Figure 3**.

## Discussion

The term “myxofibrosarcoma” was first introduced by Angervall et al. in 1977 (4). In 2002, the WHO reclassified MFS as a distinct tumor entity, thereby distinguishing it from MFH, which had previously been regarded as its counterpart (5). Histologically, MFS is characterized by curvilinear, thin-walled vessels within a prominent myxoid stroma, often surrounded by perivascular tumor cells and inflammatory cells.

The diagnosis of MFS remains challenging because of the absence of specific immunohistochemical markers, and it is therefore largely established by exclusion. Although vimentin and CD34 expression is frequently observed, these findings are nonspecific and mainly indicate mesenchymal differentiation (6). In our case, an extensive immunohistochemical panel was employed to systematically exclude histologic mimics, which is particularly critical in the intracranial setting given the considerable morphologic overlap among different tumor entities. The integration of characteristic histopathologic features with immunophenotypic findings allowed for the reliable exclusion of epithelial, myogenic, neuroectodermal, and meningeal neoplasms, as well as selected sarcoma subtypes, thereby supporting the diagnosis of primary intracranial myxofibrosarcoma.

Histopathology and molecular analyses remain essential for a definitive diagnosis, whereas MRI provides complementary but nonspecific information. On MRI, primary intracranial MFS typically appears as a solid or nodular mass with heterogeneous enhancement, which may radiologically resemble meningioma, glioblastoma, or metastatic lesions (12). In our case, the lesion was initially misinterpreted as a hemangioblastoma or glioblastoma, highlighting the diagnostic challenges and the necessity of histopathologic confirmation.

Primary intracranial MFS is exceedingly rare. To the best of our knowledge, only 6 cases involving the intracranial region have been reported to date (**Table 2**). Based on the limited reported cases, primary intracranial MFS may be prone to local recurrence, which aligns with the behavior of MFS at other anatomical sites, where local recurrence occurs in up to 60% of cases (7). Moreover, several studies have shown that locally recurrent MFS often progresses to a higher histological grade, accompanied by a concomitant increase in metastatic potential (8–10). Histologically, low-grade areas can be observed within high-grade tumors, indicating a continuum from low- to intermediate- and high-grade neoplasms (11). The previously reported case involved a 21-year-old female with a primary lesion in the left fronto-parietal lobe. During follow-up, the tumor recurred twice at the original site, with each recurrence associated with a higher histological grade (12). Our case exhibited a similar pattern of recurrence and stepwise histopathological progression.

**Table 2:** Summary of reported cases of primary intracranial myxofibrosarcoma(MFS).

Case number	Author/year	Sex/age (year)	Radiation-induced (yes/no)	Symptoms	Location	Histopathological diagnosis	Treatment	Radiologic features	LR (yes/no)	Follow-up (month)
1	Kuo J et al., 2007 (19)	M/28	Yes	Headache, drowsiness, right-sided weakness	left scalp (temporo-parietal) with intracranial extension	malignant MFS, FNCLCC grade III/III	S + RT	Irregular, heterogeneously enhancing mass with intralesional hemorrhage	N/A	N/A
2	Buccoliero AM et al., 2011 (20)	M/9	No	Nausea, sweating, confusion (1 month); recent neck/back pain, diplopia	left parieto-occipita	Low grade intracranial MFS	S + RT + C	irregular peripheral enhancement and central necrosis	Yes	15
3	Majumdar K et al., 2013 (12)	F/21	No	Two seizure episodes, mild headache, mild right hemiparesis (1 month)	left fronto-parietal lobe	MFS	S + RT	Space-occupying lesion attached to the falx and convexity dura	Yes	30
4	Costa DA et al., 2016 (21)	M/10	No	N/A	Left parieto-occipital convexity, extra-axial	Low grade MFS	S + RT	N/A	Yes	N/A
5	Sotiriou S et al., 2020 (11)	M/55	No	Movement, speech, and behavioral disturbances (1 month)	Right frontal lobe	High grade intracerebral MFS	S	mass effect, midline shift, and ring enhancement	N/A	N/A
6	Matias TB et al., 2022 (22)	M/42	No	Lethargy, right ptosis, quadriparesis	Right extra-axial frontal	Low grade intracranial MFS	S + RT + C	heterogeneous hyperintensity, heterogeneous solid peripheral enhancement, high rCBV, restricted diffusion	Yes	12

M, male; F, female; MFS, Myxofibrosarcoma; S, surgery; C, chemotherapy; RT, radiotherapy; rCBV, relative cerebral blood volume;LR, local recurrence; N/A, not available.

However, unlike previously reported cases, the recurrences in our patient involved anatomically separate lobes with distinct leptomeningeal involvement. The primary tumor was located in the right frontal lobe, accompanied by adjacent leptomeningeal enhancement. The first recurrence occurred in the right temporal lobe, within the same-side leptomeningeal region as the primary lesion. The second recurrence developed primarily in the left frontal lobe, associated with pronounced leptomeningeal thickening and enhancement. This pattern of non-contiguous intracranial recurrence is unlikely to result from direct local spread of residual tumor. Although multicentric tumors have been described in central nervous system neoplasms, such as gliomas, where spatially separated lesions without imaging continuity may arise independently, the temporal and spatial sequence of recurrences in our patient argues against a true multicentric origin (13). Considering the radiological features, cerebrospinal fluid (CSF) dissemination represents a plausible mechanism, whereby tumor cells may spread along the leptomeningeal surface or through the ventricular system to distant intracranial sites. Although CSF cytology was not performed, its sensitivity is limited and false-negative results are common in central nervous system tumors (14). Nevertheless, the presence of multiple foci of leptomeningeal enhancement on imaging provides indirect support for this hypothesis. Importantly, all recurrent lesions occurred outside the previous radiotherapy fields, which argues against a radiotherapy-induced recurrence (15).

The management of MFS is primarily surgical, with negative margins being essential to reduce the high risk of local recurrence (16). Adjuvant radiotherapy is important for improving local control and is generally recommended even for small tumors (17). Chemotherapy has limited efficacy, as most retrospective studies have either shown minimal benefit or been limited by small sample sizes (18). In this case, intracranial recurrences occurred despite multiple surgeries combined with adjuvant radiotherapy, illustrating the intrinsic high recurrence risk of MFS and the challenges in maintaining durable local control. These findings highlight the need for individualized surgical strategies, precise radiotherapy target delineation, and long-term imaging follow-up. While based on a single case, these findings suggest that patients with early leptomeningeal involvement may benefit from individualized and prolonged imaging follow-up extending beyond the primary tumor site.

## Conclusion

In summary, we report a 34-year-old male patient with primary intracranial myxofibrosarcoma who initially presented with left-sided limb numbness and later developed two non-contiguous recurrences in distinct cerebral lobes. Although preoperative imaging features mimicked a glioma or hemangioblastoma, surgical excision followed by histopathological analysis confirmed the lesion as MFS. Despite maximal surgical resection and adjuvant radiotherapy, the patient developed two recurrences within three years, underscoring the aggressive nature and propensity for local recurrence of intracranial MFS. Close follow-up is essential, and individualized comprehensive treatment strategies should be considered in cases of multiple recurrent tumors.

## Data Availability

The raw data supporting the conclusions of this article will be made available by the authors, without undue reservation.
